# Coma and Narcotism

**Published:** 1861-09

**Authors:** 


### Coma and Narcotism.

In a paper *On the Importance of Free Respiration in certain states of the Brain*—read at the Western Medical and Surgical Society, London—Dr. W. C. Hunter stated in effect that when a patient is comatose, the lungs gradually become more and more congested, effusion takes place into the pulmonary tissue, and accumulates in the bronchial tubes; yet there is no actual paralysis of the respiratory muscles. In death from narcotism, the respiratory muscles and tongue are paralyzed, but there is neither rapid effusion nor the great congestion of coma. Now, the treatment of these two cases is quite different. In the treatment of the former, venesection is our sheet-anchor, to relieve effusion and prevent its further formation, and to favor the exit of the bronchial effusion the prone position is necessary. Artificial respiration is unnecessary. In the latter, artificial respiration is invaluable, whilst venesection is uncalled for. The prone position is necessary on account of the lingual paralysis.

*Braith, Ret.*, 41.

